# Osteoma Presenting as a Painless Solitary Mastoid Swelling

**DOI:** 10.1155/2015/590783

**Published:** 2015-02-12

**Authors:** Hazem M. Abdel Tawab, Ravi Kumar V, Salim M. Sloma Tabook

**Affiliations:** ^1^Department of Otorhinolaryngology, Faculty of Medicine, Cairo University, Cairo, Egypt; ^2^Department of Otorhinolaryngology, Sultan Qaboos Hospital, Salalah, Oman

## Abstract

*Introduction.* Osteoma of the temporal bone is a very uncommon benign tumor of bone. Osteomas may occur in the external auditory canals but are reported to be very rare in the mastoid bone. *Case Report.* A 36-year-old male presented to our department with a hard swelling behind the right ear diagnosed as osteoma. Complete excision was done through a postauricular approach. Histopathology confirmed the diagnosis of osteoma. *Conclusion.* Osteomas, although rare, should be considered when dealing with any hard mastoid swelling. Complete removal can be ensured by drilling till the normal cortical bone is reached to avoid recurrence. A cortical mastoidectomy should be done if the mastoid air cell system is involved.

## 1. Introduction

Osteoma is a benign tumor of mesenchymal osteoplastic nature composed of well-differentiated osseous tissue with laminar structure [1]. Osteomas are extremely rare in the temporal bone and mostly occur in the external auditory canal, while mastoid osteomas are rarer [2]. Temporal bone osteomas in general constitute 0.1% to 1% of all benign tumors of the skull [3]. Causes of mastoid osteoma reported in the literature included trauma, previous surgery, radiotherapy, chronic infection, and hormonal factors with dysfunction in the hypophyseal gland [4]. We present a case of mastoid osteoma presenting as a single painless swelling in the postauricular region.

## 2. Case Report

**Figure 1:**
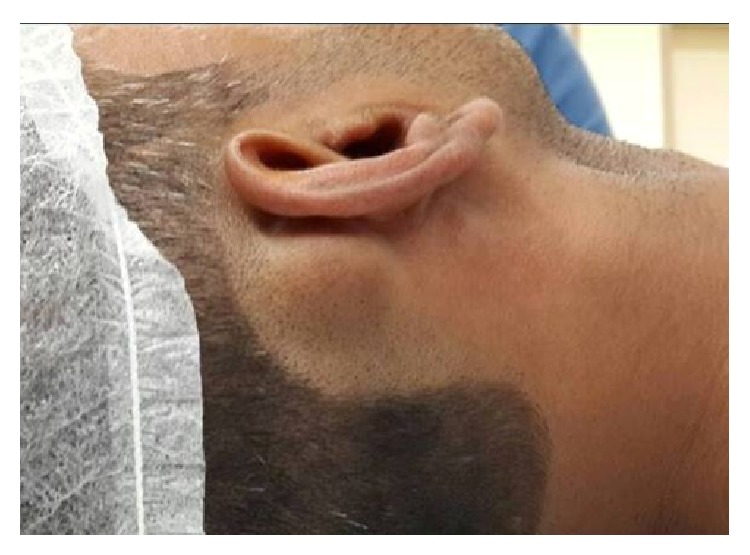
Osteoma external appearance.

**Figure 2:**
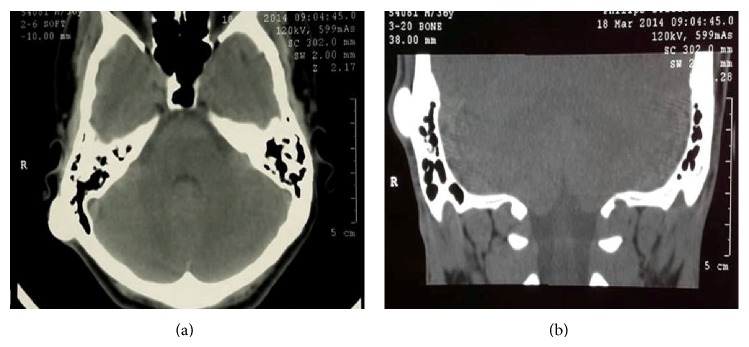
(a) Osteoma in axial cut CT scan. (b) Osteoma in coronal cut CT scan.

A 36-year-old male presented to us at Sultan Qaboos Hospital, Salalah, Oman, with a postauricular hard swelling in the right mastoid region. There was no associated pain, deafness, dizziness, or cranial nerve deficit. There was no history of scalp infection. The only complaint of the patient was the cosmetic deformity caused by this swelling for which he sought medical advice. It had been progressively becoming larger over the last seven years. On examination, there was a bony hard, sessile, and nontender swelling, 2.5 cm × 2.5 cm in dimensions (Figure 1), and the overlying skin was free. Facial nerve function was intact and no exostosis was encountered in either of the external auditory canals. No other abnormality was detected with the rest of ear, nose, and throat examination. There was no significant abnormality in the ophthalmological and the rest of systemic examination. All routine laboratory tests were normal and computed tomography (CT) of the right temporal region revealed a radiodense broad based swelling in the right mastoid and squamous temporal bone (Figure 2). No radiological abnormalities were seen in the middle and inner ears. There is no evidence of another density in the temporal region.

**Figure 3:**
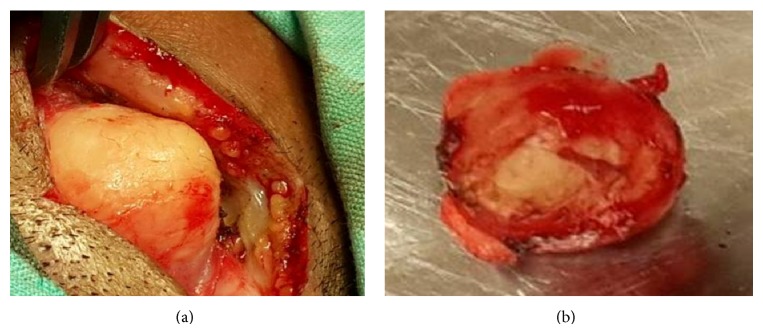
(a) Osteoma after freeing it all around before its excision. (b) The specimen after removal.

A modified postauricular incision was given to provide complete exposure of the osteoma. A groove was drilled around the base and the osteoma was chiseled out in total (Figure 3). Since the mastoid air cells were encountered in the anterior part of dissection, a cortical mastoidectomy was done to ensure complete removal and prevent recurrence. The incision was closed in layers with a drain and stitches were removed ten days after surgery with no complications in the postoperative period.

Histopathology confirmed the diagnosis of compact osteoma. At 4-month follow-up the patient was free of symptoms and there was no sign of recurrence or complications.

## 3. Discussion

Osteoma of the mastoid bone is a benign tumor that is slowly growing and circumscribed in shape [5].

D'Ottavi et al. reviewed 100 cases of mastoid osteoma and reported two cases of their own [6]. Amongst parts of the temporal bone, the external auditory canal is the most common location for osteomas, followed by the mastoid and the temporal squama [7]. The etiology of osteoma is unknown, but there is some evidence to suggest a congenital origin [8]. Embryogenesis and metaplasia following recurrent local irritation and trauma are the most commonly accepted theories for occurrence of osteoma. Osteomas usually occur singly, but when they are multiple, Gardner's syndrome must be ruled out. Osteoma may occur alone or as a part of a syndrome. Gardener's syndrome, which is characterized by multiple intestinal polyps, epidermoid inclusion cysts, fibromas of the skin, and mesentery and osteomas, must be ruled out in every single case. Osteomas in this case usually affect membranous bones as mandible and maxilla [9]. Osteomas of the skull may be compact, spongy, or mixed.

Osteomas are mostly asymptomatic, but they can present with deformity, swelling, pain, deafness, and chronic discharge [10]. The differential diagnosis includes osteosarcoma and osteoblastic metastasis.

CT scanning is the imaging method of choice for mastoid and all other osteomas. The imaging appearance reflects the underlying pathology, with ivory osteomas appearing as very radiodense lesions, similar to normal cortex, whereas mature osteomas may demonstrate central marrow. It is seen as a high opacity, well-demarcated, and dense growth of sclerotic lesion from the mastoid bone [1].

Mastoid osteomas are resected if symptomatic or for cosmetic reasons. The excision should be complete till the normal cortical bone is reached all around. If mastoid air cells are exposed, a cortical mastoidectomy should be done [11].

In the present case, the marked swelling, causing deformity, was the reason of excision. Because these lesions are limited to the external cortex, finding a plane of cleavage between the osteoma and normal bone is not difficult [12]. In the case presented, cortical mastoidectomy was done due to close attachment of the tumor to the mastoid cortex and its wide base.

The prognosis of such tumors is good. Due to the fact that mastoid osteoma may be a part of Gardner's syndrome, it should be followed carefully. Gardner's syndrome should be excluded in every case by doing ophthalmological examination or colonoscopy. Recurrence is rare and malignant transformation has not been reported [11].
